# A Ras-LSD1 axis activates PI3K signaling through PIK3IP1 suppression

**DOI:** 10.1038/s41389-019-0185-4

**Published:** 2020-01-02

**Authors:** Kyunghee Lee, Mayumi Kitagawa, Pei Ju Liao, David M. Virshup, Sang Hyun Lee

**Affiliations:** 1 Programme in Cancer and Stem Cell Biology, Duke-NUS Medical School, Singapore, 169857 Singapore; 2 Prestige Biopharma Pte Ltd, Singapore, 117543 Singapore; 3 MK, Daiichi Sankyo Co., Tokyo, 103-8426 Japan; 4 Department of Pediatrics, Duke University School of Medicine, Durham, NC 27710 USA

**Keywords:** Cancer, Epigenetics

## Abstract

PI3K Interacting Protein 1 (PIK3IP1) is a suppressor of the PI3K/Akt/mTOR pathway. We previously reported that activated Ras suppresses PIK3IP1 expression to positively regulate the PI3K pathway in cancer cells. Using doxycycline-inducible PIK3IP1, here we confirm that reversing the effect of Ras by inducing expression of PIK3IP1 suppresses Ras-induced anchorage-independent growth, supporting the central role of PIK3IP1 in transformation. However, the molecular mechanisms by which Ras-activation that causes loss of PIK3IP1 expression are unknown. We find that Ras activity represses PIK3IP1 expression via the recruitment of lysine-specific demethylase 1 (LSD1) to the PIK3IP1 gene promoter and enhancer, resulting in erasure of active histone marks. These studies demonstrate cross-activation of Ras/Raf/MEK/ERK and PI3K/AKT/mTOR pathways, where Ras decommissions PIK3IP1 gene expression by enhancing LSD1 and its corepressor activities to suppress PIK3IP1 transcription.

## Introduction

The PI3K/AKT/mTOR and Ras/Raf/MEK/ERK signaling pathways transmit signals from receptor tyrosine kinases (RTKs) to downstream effector networks regulating cell growth, survival, and proliferation in response to external cues^1,2^. The high frequency of mutations in these pathways in multiple types of cancer has led to the development of selective inhibitors for cancer therapy. Data from cancer genomics for representative components of each pathway have revealed that K-Ras and PIK3CA, K-Ras and Phosphatase and tensin homolog (PTEN) or B-RAF and PTEN are mutually exclusive in lung, urine, and colorectal cancers (http://www.cbioportal.org). Nevertheless, several feedback mechanisms that cross-talk or cross amplification of signaling events occurs between these pathways can render tumor cells resistant to therapy. However, the molecular components that mediate resistance and cross-talk between these two central pathways are not fully understood^3^.

Phosphatidylinositol-3-kinase Interacting Protein 1 (PIK3IP1) has been characterized as a new negative regulator of PI3K/Akt/mTOR pathway, which binds to the p110 catalytic PI3K subunit and leads to inhibition of the PI3K/Akt/mTOR pathway^4^. PIK3IP1 dysregulation that subsequently contribute to carcinogenesis^4–7^. We recently reported that a novel compound, named a131, highly increases the PIK3IP1 expression and this causes the PI3K/Akt/mTOR pathway inhibitions in normal BJ-H-RasV12-ER cells but not in transformed counterparts. However, 4-HT-induced H-RasV12-ER activation is sufficient to reactivate the PI3K/Akt/mTOR pathway through downregulating mRNA and protein levels of PIK3IP1 in a131-treated normal BJ-H-RasV12-ER cells. In contrast, pharmacological inhibition of MAPK activity attenuates Ras-induced PIK3IP1 suppression. In addition, the PIK3IP1 mRNA levels are significantly lower in Ras/Raf-mutant cancer cells. Using Ras-activated cancer cells and clinical samples from patients with colorectal and lung adenocarcinomas, we have described the underlying mechanism for cross-activation, positive cross-talk, between the Ras/Raf/MEK/ERK and PI3K/AKT/mTOR pathways through PIK3IP1 suppression^8^. However, the regulatory mechanism underlying PIK3IP1 gene suppression by Ras in cancer cells remains unknown.

Epigenetic silencing of gene expression plays a major role in cancer development. Aberrant DNA CpG island hyper-methylation and histone modifications can lead to silencing of tumor suppressor genes^9,10^. Lysine-specific demethylase 1 (LSD1, also known as KDM1A) is the first discovered flavin-dependent histone demethylase^11^. LSD1 recruitment to target gene with the REST corepressor (CoREST) can result in transcriptional repression by demethylating H3K4, where LSD1 removes active marks associated with gene activation, leading to repression of the target genes. Alternatively, LSD1-mediated demethylation of H3K9 eliminates a repressive mark, resulting in transcriptional activation^12–14^. Many studies have shown that LSD1 overexpression promotes cancer cell proliferation, invasion, and metastasis^15–17^. The overexpression of LSD1 is correlated with poor prognosis in a variety of cancers and its role in transcriptional repression suggests a key role in human cancers^18^. Therefore, a number of small molecule inhibitors have been developed that target LSD1 as a potential therapy^12,14^.

Our previous study showed that active Ras inhibited the expression of PIK3IP1, a potent inhibitor of the PI3K pathway. Here we further elucidate the mechanism. Conditionally expression of PIK3IP1 confirmed that it is a critical tumor suppressor when Ras is activated. We found that LSD1 mediates PIK3IP1 suppression following oncogenic Ras activation, via the MEK/ERK pathway. Pharmacological inhibition of LSD1 restored PIK3IP1 expression in multiple Ras- or Raf-mutant cancer cells. This provides a mechanistic link between Ras activation and reactivation of PI3K signaling in cancer and further supports efforts to pharmacologically target LSD1.

## Results

### Conditional overexpression of PIK3IP1 suppresses Ras-induced transformation

**Fig. 1:**
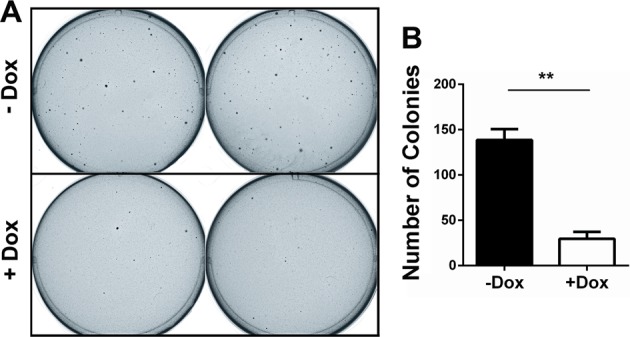
Doxycycline (Dox)-induced PIK3IP1 suppresses colony formation of A549 harboring K-Ras mutation. **a** Soft agar colony formation assay was performed in triplicate using A549 cell lines engineered to inducibly express PIK3IP1. Colonies were photographed and counted after 14d. **b** The number of colonies in Dox-treated cells was significantly reduced compared to that in untreated cells (**p < 0.01).

We previously found that *PIK3IP1* mRNA expression level was significantly downregulated in most Ras/Raf-mutant cancer cells^8^. When Ras was induced by adding 4-HT in normal BJ-H-RasV12-ER cell line, PIK3IP1 expression was significantly suppressed^8^. To test if restoring PIK3IP1 expression reversed the effects of Ras, we transduced PIK3IP1 into A549 Ras-mutant cancer cell using a Lenti-X Tet-On Inducible Expression system. Colony formation assay showed that when PIK3IP1 was conditionally overexpressed, Dox-treated A549 cells formed significantly fewer colonies compared with those without Dox treatment (Fig. 1a, b). This suggests that PIK3IP1 inhibits the anchorage-independent growth of Ras-transformed cells and PIK3IP1 suppression is a critical function of activated Ras.

### Ras downregulates *PIK3IP1* promoter activity

**Fig. 2:**
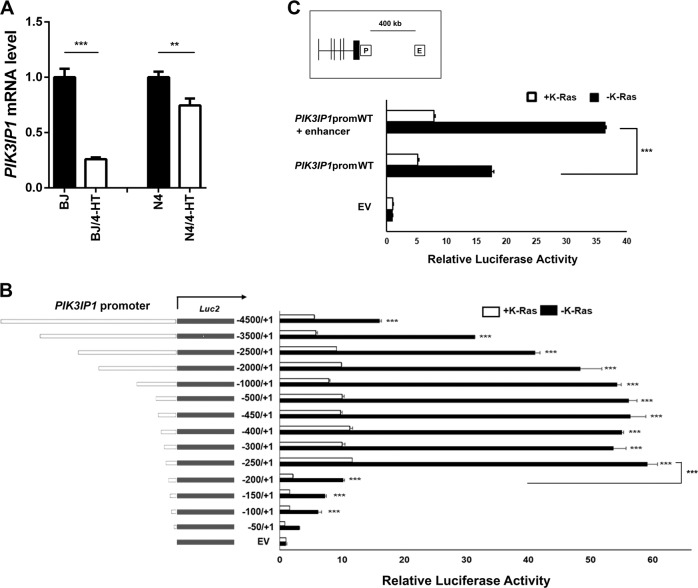
*PIK3IP1* promoter is inactivated by Ras. **a** mRNA abundance of *PIK3IP1* in BJ-H-RasV12-ER and N4-H-RasV12-ER cells with or without 4-HT treatment was assessed by RT-qPCR. *PIK3IP1* expression without 4-HT (Black bar) was designated as 1, and the value with 4-HT treatment (white bar) was normalized to this. **b** Dissection of the Ras-regulated *PIK3IP1* promoter. The indicated genomic fragments starting 4500 bp upstream of the PIK3IP1 transcriptional start site were cloned into pGL4.10 Empty vector (EV) served as a negative control (EV). Reporter constructs were co-transfected with either control or K-Ras expression vector. Open boxes correspond to the promoter region upstream of the transcription start site. **c** Comparison of *PIK3IP1* transcriptional activity in *PIK3IP1* promoter constructs and promoter construct with the distal enhancer. Data were presented as the means ± SD of three independent experiments. **p < 0.01, ***p < 0.001 and *ns*, not significant by Student’s *t*-test as compared to control cells.

To identify the mechanism by which Ras activation suppressed PIK3IP1 expression, we used isogenic human BJ-H-RasV12-ER and N4-H-RasV12-ER cells (For detailed information, referred to “Materials and Methods”). We first analyzed mRNA levels of *PIK3IP1* both in BJ-H-RasV12-ER and N4-H-RasV12-ER cells. As we previously reported^8^, 4-HT-induced H-Ras activation was sufficient to downregulate *PIK3IP1* mRNA levels in both BJ-H-RasV12-ER and N4-H-RasV12-ER cells (Fig. 2a). Next, to examine the promoter activity of *PIK3IP1* under Ras activation, we performed luciferase reporter assays by systematically deleting the *PIK3IP1* promoter region (~4.5 kb) from the 5’ end (Fig. 2b). The full length and deletion mutants of the *PIK3IP1* promoter were cloned into pGL4 Basic vector bearing firefly luciferase cDNA, and each construct was co-transfected with either K-Ras expression or empty vector into 293 T cells. *PIK3IP1* promoter by itself (black bars) displayed a strong transcriptional activity (Fig. 2b). However, in cells co-transfected with K-Ras expression vector (white bars), the basal luciferase activity was reduced by 6-fold when compared with K-Ras empty vector co-transfection (black bars) (Fig. 2b). These data suggest that Ras activation is responsible for repression of *PIK3IP1* transcriptional activity. Moreover, the deletion series of the *PIK3IP1* promoter showed that basal luciferase activity reached its maximum from −500 bp and it was 3-fold greater than that of the full-length promoter (4.5 kb). However, the *PIK3IP1* promoter activity dropped significantly (*p* < 0.001) when the promoter was deleted from its 5’ end to −200 bp and it became undetectable level when the promoter was deleted to −50 bp (Fig. 2b). Further, as shown in Fig. S1, the 150 bp region located between −250/−100 was both necessary and sufficient for maximal promoter of the *PIK3IP1* gene. This promoter structure will provide further insight into the minimal requirements for Ras regulation of the *PIK3IP1* promoter.

The UCSC Genome Browser-predicted PIK3IP1 enhancer (chr22:31689398–31689773) is over 400 kb away from the PIK3IP1 transcription start site. The construct including this enhancer had 2-fold increased activity compared to the construct lacking it, suggesting that this putative sequence has a role as an enhancer and helps increase expression of *PIK3IP1* (Fig. 2c).

### Oncogenic Ras signaling diminishes histone active marks at *PIK3IP1*’s enhancer and promoter sites

**Fig. 3:**
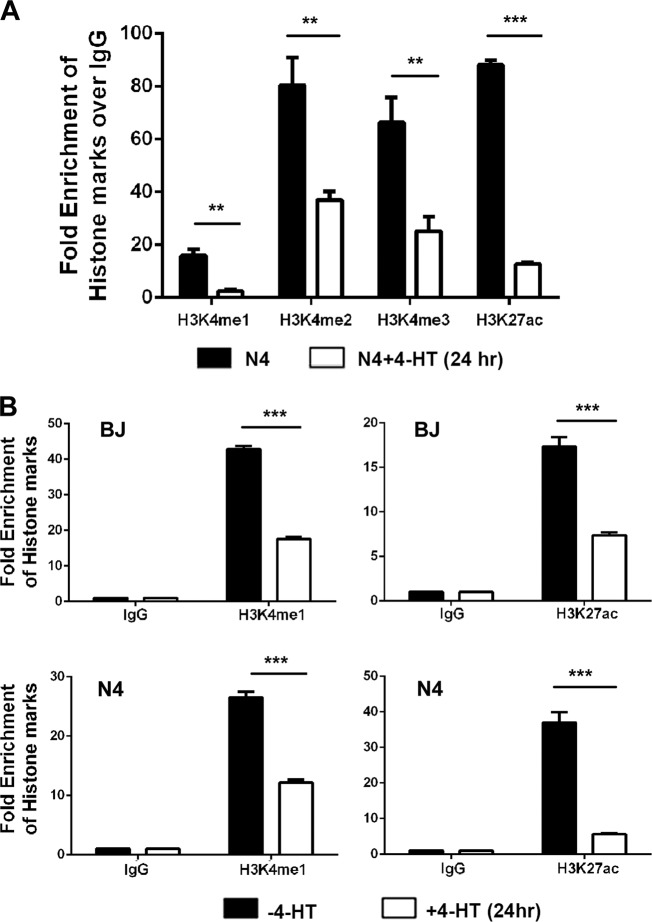
Ras activation using 4-HT suppresses histone activation marks on the *PIK3IP1* promoter and enhancer. **a** ChIP-qPCR assay of *PIK3IP1* promoter with indicated histone mark antibodies in N4-H-RasV12-ER cells treated with or without 4-HT for 24 hr. **b** ChIP-qPCR assay of UCSC Genome Browser-predicted *PIK3IP1* enhancer with indicated histone mark antibodies after 24 hr treatment of 4-HT in BJ-H-RasV12-ER and N4-H-RasV12-ER cells. Data were presented as the means ± SD of three independent experiments. **p < 0.01 and ***p < 0.001 by Student’s *t*-test as compared to control cells.

We speculated that Ras activation might suppress PIK3IP1 expression by epigenetic modification of its promoter. We first tested if histone modifications were maintained following Ras activation with ChIP-qPCR. Treatment of N4-H-RasV12-ER cells with 4-HT for 24 h significantly reduced the histone active marks, such as H3K4me1, H3K4me2, H3K4m3, and H3K27ac, at PIK3IP1 promoter sites (Fig. 3a). Both H3K4me1 and H3K27ac marks at the PIK3IP1 enhancer were also significantly reduced in BJ-H-RasV12-ER and N4-H-RasV12-ER cells following activation of Ras by treatment with 4-HT for 24 h (Fig. 3b). Collectively, our data suggest that PIK3IP1 downregulation following Ras activation is closely linked to the decreased active histone marks at both its enhancer and promoter regions.

### Activated Ras-mediated upregulation of LSD1 is required for transcriptional silencing of *PIK3IP1*

**Fig. 4:**
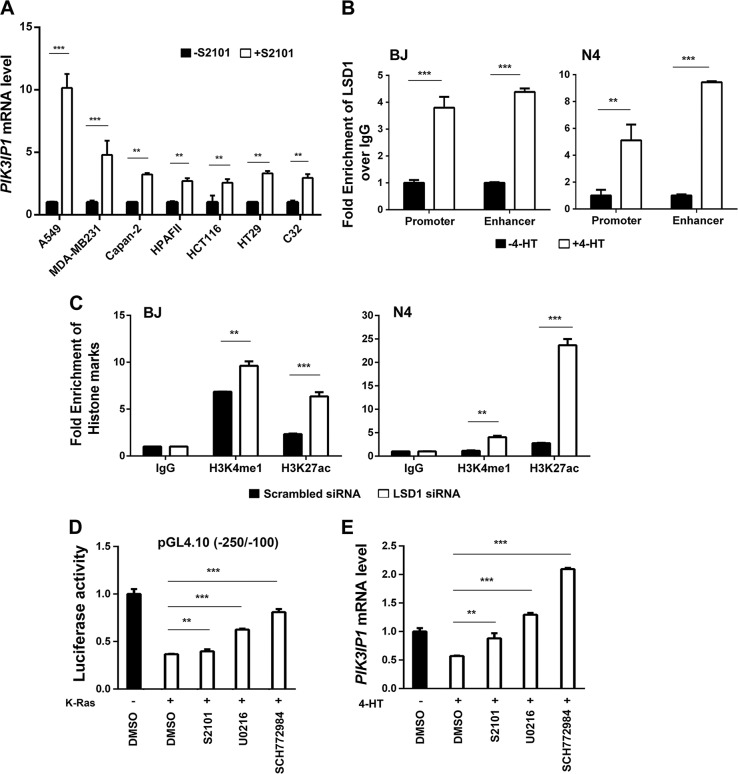
Ras activation using 4-HT increases LSD1 on PIK3IP1 enhancer and promoter regions of both BJ-H-RasV12-ER and N4-H-RasV12-ER cells. **a** Pharmacological inhibition of LSD1 with S2101 promoted *PIK3IP1* gene expression in multiple Ras/Raf-mutant cancer cell lines. *PIK3IP1* mRNA abundance in each cell line without treatment (black bars) was set to 1. **b** *LSD1* binding to the promoter and enhancer of *PIK3IP1* was enhanced by Ras activation. BJ-H-RasV12-ER and N4-H-RasV12-ER cells were treated with 4-HT for 24 h and then examined by *LSD1* ChIP-qPCR. LSD1 without 4-HT (Black bar) was set to 1. **c** *LSD1* knockdown increased activating marks at the *PIK3IP1* enhancer. ChIP-qPCR analysis of indicated histone marks was performed in BJ-H-RasV12-ER and N4-H-RasV12-ER cells after *LSD*1 knockdown. **d** *PI3KIP1* promoter activity regulated by inhibitors of MEK, ERK, and LSD1. 293 T cells were co-transfected with pGL4.10-Luc (−250/−100), minimal regulatory region of promoter, and either K-Ras expression or K-Ras empty plasmids. Twenty hour after transfection, K-Ras co-transfected cells were treated with the indicated compounds. After 8 h, the cells were collected and assayed for luciferase activity. Values were normalized against luciferase activity of DMSO treated cells co-transfected with K-Ras empty plasmids. The experiments were performed in triplicate. **e** Relative *PIK3IP1* mRNA expression by qPCR analysis in BJ-H-RasV12-ER cells were treated the indicated compounds with or without 4-HT for 24 h. PIK3IP1 expression without 4-HT was set to 1. Inhibitors and their concentrations used in **d** and **e**: LSD1 inhibitor (S2101, 20 μM), MEK inhibitor (U0126, 10 μM), and ERK inhibitor (SCH772984, 1 μM). Data were presented as the means ± SD of three independent experiments. **p < 0.01 and ***p < 0.001 by Student’s *t*-test as compared to control cells.

The decrease in active histone marks at the promoter and enhancer of *PIK3IP1* in Ras-activated cells had us lead to identify histone methyltransferases or demethylates as a target. Because of the strong correlation between these two epigenetic regulators and cancer developments^19,20^, we first tested the mRNA expression levels of *LSD1* and *KMT2C* after BJ-H-RasV12-ER and N4-H-RasV12-ER cells were treated with 4-HT. *LSD1* but not *KMT2C* mRNA expression increased significantly in response to Ras activation in BJ-H-RasV12-ER and N4-H-RasV12-ER cells (Fig. S2). To further investigate the functional relationship between PIK3IP1 and LSD1, histone demethylases, in Ras-activated cancer cells, we examined if *PIK3IP1* mRNA expression was affected following treatment of cells with the LSD1 inhibitor S2101. Following S2101 exposure the *PIK3IP1* mRNA expression level was upregulated from 3–10-fold in multiple Ras- or Raf-mutant cancer cells (Fig. 4a). This indicates that *PIK3IP1* expression is highly sensitive to LSD1 inactivation. To test if LSD1 was directly involved in regulating PIK3IP1, we performed ChIP-qPCR for *LSD1* at the *PIK3IP1* promoter and enhancer in BJ-H-RasV12-ER and N4-H-RasV12-ER cells treated with or without 4-HT. 4-HT induction of Ras activity significantly increased *LSD1* binding to the *PIK3IP1* promoter and enhancer in both cell lines (Fig. 4b). Conversely, *LSD1* knockdown by siRNA in BJ-H-RasV12-ER and N4-H-RasV12-ER cells lead to significant increases in both H3K4me1 and H3K27ac marks at the *PIK3IP1* enhancer (Fig. 4c). Finally, activity from the minimal (Fig. 4d) and endogenous (Fig. 4e) *PIK3IP1* promoter was suppressed by Ras activation, but partially rescued by LSD1 or MEK/ERK inhibition (white bars) (Fig. 4d, e). This path appears to be functional even in the absence of *RAS* overexpression, as inhibition of either MEK, ERK, or LSD1 activated the *PIK3IP1* promoter in cells without RAS hyperactivation (Fig. S3). Taken together, our data indicates that the MEK/ERK pathway stimulates LSD1 to bind to the enhancer and promoter of *PIK3IP1*, suppressing its transcription and that mutant RAS substantially increases this suppression.

**Fig. 5:**
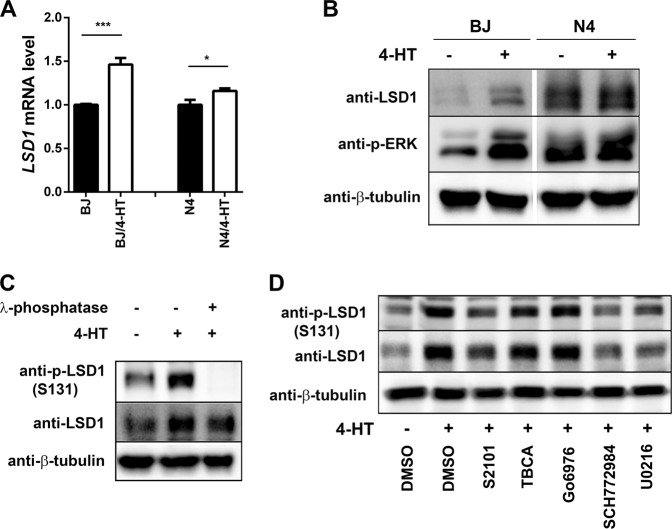
MEK/ERK pathway is necessary for the induction of LSD1 expression and activity in response to Ras. **a** Relative *LSD1* mRNA abundance in BJ-H-RasV12-ER and N4-H-RasV12-ER cells was assessed by qPCR 24 hr after treatment with 4-HT. **b** LSD1 protein abundance and ERK activation was assessed by immunoblotting in BJ-H-RasV12-ER and N4-H-RasV12-ER cells following treatment with 4-HT as above. β-tubulin was used as a loading control. **c** Ras activation increased by 4-HT treatment and λ-phosphatase treatment of lysates subsequently abolished 4-HT induced phosphorylation of LSD1 in BJ-H-RasV12-ER cells as assessed by immunoblot analysis with anti-phospho-LSD1 (S131) and LSD1 antibodies. **d** Analysis of modifiers of LSD1 phosphorylation status in BJ-H-RasV12-ER cells. LSD1 inhibitor (S2101, 20 μM), CK2 inhibitor (TBCA, 20 μM), PKCα inhibitor (Go6976, 10 μM), ERK inhibitor (SCH772984, 1 μM), and MEK inhibitor (U0126, 10 μM). Data were presented as the means ± SD of three independent experiments. *p < 0.05 and ***p < 0.001 by Student’s *t*-test as compared to control cells.

We next examined how Ras signaling increased LSD1 activity. We identified three cooperating mechanisms. First, *LSD1* mRNA increased up to 1.5-fold following Ras activation with 4-HT treatment (Fig. 5a). Second, Ras activation lead to a modest increase in LSD1 protein (Fig. 5b). Finally, Ras activation led to an increase in site-specific LSD1 phosphorylation at S131 (Fig. 5c) that was blocked by inhibitors (U0216 and SCH772984) of MEK and ERK (Fig. 5d). This suggests that LSD1 activity is upregulated by diverse mechanisms following Ras activation. While LSD1 phosphorylation has been attributed to casein kinase 2^21^ and PKCα^22,23^, in our system there was no effect of inhibitors (TBCA and Go6976) of these two kinases on S131 phosphorylation (Fig. 5d).

### PI3K/AKT/mTOR pathway is inactivated by LSD1 inhibition upon conditional Ras activation

**Fig. 6:**
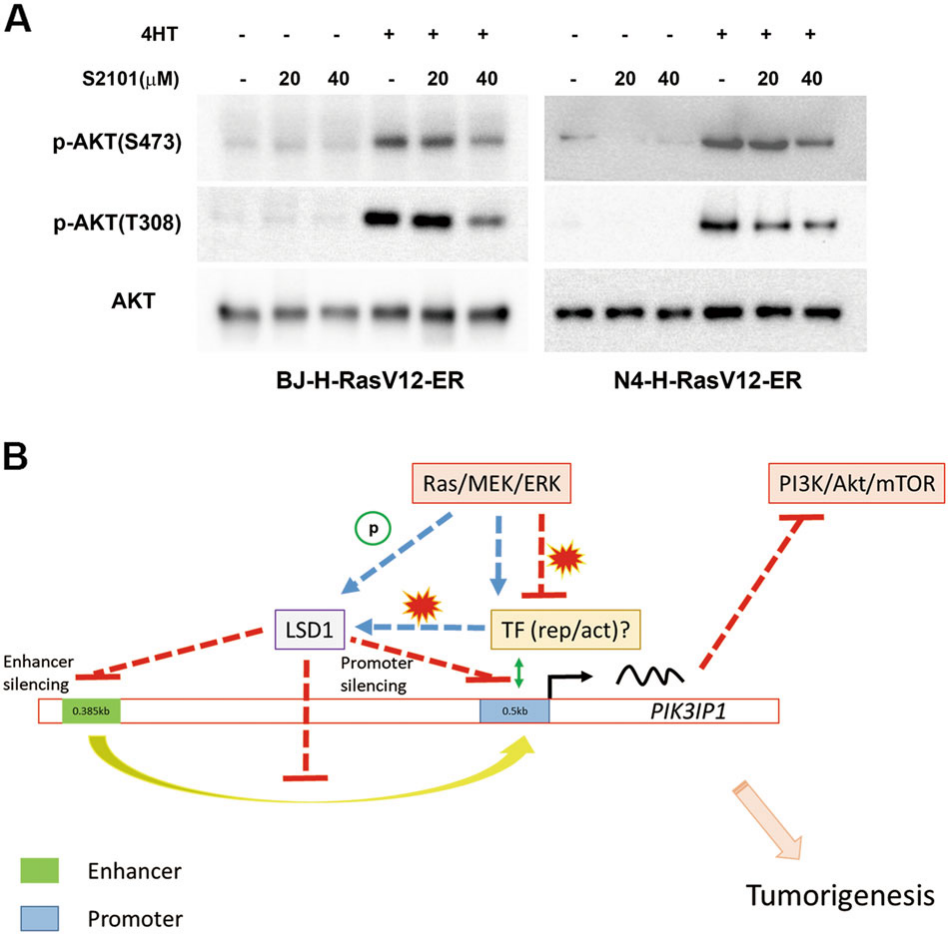
LSD1 inhibition supresses PI3K signalling upon conditional Ras activation. **a** Phosphorylation levels of AKT at both Ser473 and Thr308 in response to LSD1 inhibitor treatment (20 or 40 μM) were assessed by immunoblotting in BJ-H-RasV12-ER and N4-H-RasV12-ER cells following treatment with/without 4-HT. **b** A proposed model of Ras-induced LSD1 overexpression and phosphorylation in PIK3IP1 silencing that drives the cross-activation of PI3K pathway in Ras-mutant cancer cells.

PIK3IP1 is a negative regulator of the PI3K pathway and our data showed that LSD1 inhibition using a specific inhibitor, S2101, led to an increase of *PIK3IP1* mRNA expression in Ras-activated or Ras-transformed cells (Fig. 4a, d, e). Therefore, we predicted that LSD1 inhibition would cause inactivation of the PI3K pathway. LSD1 has been reported to activate PI3K/AKT/mTOR pathways through transcriptional regulations in cancers^24,25^. To examine the effect of LSD1 inhibition on PI3K signaling, BJ-H-RasV12-ER and N4-H-RasV12-ER were treated with either 20 µM or 40 µM LSD1 inhibitor (S2101) in the absence or presence of 4-HT for 24 h. AKT activation was assessed by western blotting with two phospho-AKT antibodies (S473 and T308). AKT activity was increased by 4-HT treatment in both cell lines, and was suppressed by LSD1 inhibition by S2101 in a concentration dependent manner (Fig. 6a). This result suggests that LSD1 activates the PI3K pathway through *PIK3IP1* transcriptional suppression and provide functional evidence for the role of LSD1 in repression of PIK3IP1.

In summary, oncogenic Ras through the MEK/ERK pathway activates LSD1 and increases the binding of the LSD1 corepressor complex to the *PIK3IP1* promoter and enhancer to decommission PIK3IP1 activity, which in turn reactivates the PI3K pathway in Ras mutated cancer (Fig. 6b).

## Discussion

We previously demonstrated that a novel compound, a131 selectively kills Ras-mutant cancer cells. a131 is not lethal to normal cells, where it instead causes a reversible growth arrest by transcriptionally upregulating the PI3K inhibitor, PIK3IP1. The selectivity of a131 for Ras-mutant cancer cells is due to the ability of activated Ras to suppress *PIK3IP1* transcription^8^. Here we dissect the mechanism of that suppression. We found that Ras directly regulates transcriptional activity of PIK3IP1 and promoter construct with the distal enhancer exhibits higher promoter activity. We identify the epigenetic modifier LSD1 as a critical mediator between the Ras signaling pathway and PIK3IP1 inactivation. LSD1 in a complex with CoREST and histone deacetylase 1/2 (HDAC1/2) catalyses the removal of methyl groups on H3K4 and promotes transcriptional suppression of downstream target genes^12–14^. LSD1 accumulated at both the enhancer and promoter regions of PIK3IP1 following Ras activation (Fig. 4). Collectively, our findings indicate that Ras decommissions PIK3IP1 through the epigenetic regulator LSD1. There may be additional mechanisms to control PIK3IP1 downregulation upon Ras activation. For example, Ras signaling controls the activity of the Forkhead box O (FOXO) family of longevity-related transcription factors^26^ and this may also allow LSD1 to maintain repression of the PIK3IP1.

LSD1 activity can be regulated at multiple levels, and our data suggest that several mechanisms play a role in suppression of PIK3IP1. Notably, we find a robust increase in the phosphorylation of LSD1 as well as increases in its protein abundance. The Ras-driven phosphorylation of LSD1 required the activity of MEK and ERK, but it is not clear these kinases are directly phosphorylating LSD1. While prior studies have found that LSD1 is phosphorylated by protein kinase CK2^21^ and PKCα^22,23^, in our hands inhibitors of these kinases had no effect on LSD1 phosphorylation (Fig. 5d). The role of phosphorylation of LSD1 in the suppression of PIK3IP1 remains enigmatic, as mutation of multiple putative phosphorylation sites did not substantially abrogate its activity (Fig. S4).

LSD1 is composed of three functional domains: SWIRM (residues 172–270), amine oxidase (AOL domain, residues 271–417 and 523–833), and Tower domains (residues 418–522) consisting of a large insertion in the AOL domain^27^. The N-terminus of the SWIRM domain has been reported to play a role in maintaining protein stability and the Tower domain, C-terminal of LSD1, is indispensable for the demethylase activity and interaction with CoREST^27,28^. In some the demethylase activity of LSD1 is not essential for its function. For instance, the physical interaction of LSD1/CoREST with the SNAG domain transcription repressor GFI1 was found to be required to maintain widespread gene repression, but enzymatic activity was not required^29^. However, our ChIP-qPCR and LSD1 inhibition data using the specific inhibitor S2101 that targets enzymatic function indicated that activated Ras increases LSD1 demethylase activity at both PIK3IP1’s enhancer and promoter regions and this activity has a role in epigenetic PIK3IP1 regulation. It remains unclear if phosphorylation-induced activation of LSD1 is indeed a key part of this mechanism. Thus far, we have only examined the phospho-mutants at the N-terminal (Fig. S4). Our results suggest that LSD1 phosphorylation under Ras activation can occur through multiple phosphorylation sites. Some of these potential site(s) may be located at the C-terminal. Future studies are warranted to elucidate whether LSD1-associated phosphorylation site(s) are also in the C-terminal.

In summary, we have revealed a cross-activation pathway between Ras/Raf/MEK/ERK and PI3K/AKT/mTOR signaling via PIK3IP1 suppression. Our findings provide further insight into the role of this signaling network in regulating cross-talk between the two pathways that are implicated in treatment resistance observed in cancer patients. One critical implication of this study is that LSD1 is required to silence its target gene, PIK3IP1. Therefore, as shown in Fig. S5, inhibiting both LSD1 and Ras pathway can be used as potential targeted therapy in PIK3IP1-inactivated or Ras-activated tumors.

## Materials and methods

### Cell lines and siRNA transfection

Isogenic human BJ foreskin fibroblasts that were immortalized with either hTert only (hereafter referred as BJ) or fully transformed with hTert, small t, shRNAs against p53 and p16 (hereafter referred as N4) and H-RasV12-ER (estrogen receptor-fused H-Ras bearing the activating G12V mutation) were kind.pngts from Dr. Mathijs Voorhoeve^30^. Human embryonic kidney HEK293T and all cancer cell lines used in this study were purchased from ATCC and tested for mycoplasma infection. Cell lines were maintained in DMEM (Invitrogen, Carlsbad, CA, USA) supplemented with 10% FBS (Hyclone, Logan, UT, USA), penicillin (100 U/mL), and streptomycin (100 mg/mL, Invitrogen) at 37 °C with 5% CO_2_. H-RasV12-ER was activated by exposing the BJ-derived fibroblasts to 4-Hydroxytamoxifen (4-HT,100 nM, Sigma, St. Louis, MO, USA). SMARTpool siRNA (L-009223–00–0010, Dharmacon, Lafayette, CO, USA) was used to knockdown human LSD1. Nonsilencing control siRNA (scrambled siRNA) was purchased from Dharmacon. Lipofectamine 2000 (Invitrogen) was used for siRNA transfection according to the manufacturer’s instructions.

### Inducible A549 cell line construction

Stable cell line with tetracycline-inducible PIK3IP1 (A549/Tet-On/PIK3IP1) were generated by co-transducing the A549 cells with the lentivirus system (Clontech, Mountain View, CA, USA) derived from the pLVX-Tet-On advanced regulator and the pLVX-Tight-Puro with PIK3IP1 and selected with 1 µg/ml puromycin. Doxycycline (1.25 µg/ml) was added into media to induce PIK3IP1. The concentration of Dox was confirmed by A549 cell growth assay in the absence or presence of this concentration.

### Reagents

U0126 (Cat. # 1144, MEK inhibitor) and Go6976 (Cat. # 2253/1, PKCα inhibitor) were purchased from TOCRIS (Bristol UK). TBCA (Cat. # 934358–00–6, CK2 inhibitor) and S2101 (Cat. # 48977, LSD1 inhibitor) were purchased from Calbiochem (San Diego, CA, USA). SCH772984 (Cat. # CT-SCH772, ERK inhibitor) and Tamoxifen were purchased from Chemie Tek (Indianapolis, IN, USA) and Sigma, respectively. Dual-Luciferase Reporter Assay system was purchased from Promega (Madison, WI, USA).

### RNA isolation and quantitative PCR (qPCR)

Total RNA was isolated using RNAeasy mini kit (Qiagen, Hilden Germany). cDNA synthesis was performed using 1 µg of total RNA using iScript cDNA synthesis kit (BIO-RAD, Hercules, CA, USA). qPCR analysis was performed using the iQ SYBR Green Super mix (BIO-RAD) with the following gene-specific primers: human PIK3IP1 (FW 5’-GCTAGGAGGAACTACCACTTTG-3’, REV 5’-GATGGACAAGGAG CACTGTTA-3’) and human LSD1 (FW 5’-CAGGCTTGGCAGCAGCTCGA-3, REV 5’-TCCACCCACACGATCCCTGGC-3’). The TATA binding protein (TBP) gene was used for normalization. All PCR reactions were performed in triplicate.

### Chromatin immunoprecipitation (ChIP) assays

Both BJ-H-RasV12-ER and N4- H-RasV12-ER cells were cultured in the absence or presence of 4-HT for 24 h. They were then treated with 1% formaldehyde to cross-link the DNA and lysed. ChIP assays were carried out using the Magna ChIP A/G Kit (Millipore, Burlington, MA, USA) according to the manufacturer’s instructions. ChIP DNA samples were analyzed by qPCR using iQ SYBR Green Super mix (Bio-Rad). Antibodies used in the assays were anti-H3K4me1 (Cat. # 61633, Active Motif, San Diego, CA USA), anti-H3K4me2 (Cat. # 39914, Active Motif), anti-H3K4me3 (07–473, Millipore), anti-H3K27ac (Cat. # 39133, Active Motif), LSD1 (ab17721, Abcam, Cambridge UK), normal mouse IgG (12–371, Millipore) and rabbit IgG (12–370, Millipore). The following ChIP primer sets were used for the ChIP assay: *PIK3IP1* promoter at −224/−110 (FW 5’-ACCTAGGAGGCTTAGGACCC-3’, REV 5’-CAGTCAGACCTGCCCTTGTT-3’) and UCSC Genome Browser-predicted *PIK3IP1* enhancer (chr22:31689398–31689773), ~400 kb upstream of PIK3IP1 transcriptional start site, (FW 5’-CAGTGGCTCAAACATGGCTC-3’, REV 5’-GAGCCATGTTTGAGCCACTG-3’). The ChIP enriched DNA levels were then normalized to input DNA, followed by subtraction of non-specific binding resulted from control IgG antibodies. All experiments were performed in triplicate.

### Construction of *PIK3IP1* promoter constructs and promoter construct with the distal enhancer

pGL4.10-PIK3IP1promWT constructs (4.5 kb) containing the upstream region of the PIK3IP1 transcriptional start site were kind.pngts from Dr. D. J. Adams^6^. A series of deletion constructs was generated using pGL4.10-PIK3IP1prom WT as a template. Forward primers sequences used were:

5-GAAGGCTACACTTCAAAGAAACAGCCTTGGTG-3’ (−4500/+1),

5’-AGTACAAGTGCTGGCCTGTTATAGCCTGTCTC-3’ (−3500/+1),

5’-TGTTGCCCAAGCTGGAGTGCAGTGGCATGATC-3’ (−2500/+1),

5’-TCTTCCCTGATAAAGCTTTTAAAATTTATTTA-3’ (−2000/+1),

5’-AACACTTTGGGAGGCCGAGGCAGGTGGATTAC-3’ (−1000/+1),

5’-CCACCAGAAACTCTTATTTTTTCCCCCCTATG-3’ (−500/+1),

5’-CTGGGTGCATAGCTCCCTAAGTTGTTCTGTGT-3’ (−450/+1),

5’-ACATCCCATGATGCCCCTTGATAGAATTATTT-3’ (−400/+1),

5’-AACTGATCGGCTGCTAAGCACAACCTGCATAT-3’ (−300/+1),

5’-TCTTGGCTCAGTTAGATTTTGCATGACCTAGG-3’ (−250/+1),

5’-GCGCCTTTCAGCTGAAAAACAGCTCGCGCTGC-3’ (−200/+1),

5’-TCCCAGTTCTAAAGAGAGGCTGTTTACCAGAA-3’ (−150/+1),

5’-GTCTGACTGCAAGGCTGGGACTGGGAGGCAGA-3’ (−100/+1) and

5’-TCGGTTAAACACTGGTCGTTCAATCACCTGCA-3’ (−50/+1).

One reverse primer, 5’-CCTTGCCTCCTTCGTCTTGCAGGTGATTGAACG-3’ was used for all 5’ deletion constructs. All PCR products were digested with SacI and XhoI and cloned into pGL4.10 [luc2] (Promega). For preparation of pGL4.10-PIK3IP1promWT with enhancer, genomic fragments corresponding to possible enhancer regions (chr22:31689398–31689773) were PCR amplified and cloned into pGL4.10-PIK3IP1promWT constructs using SacI enzyme. Forward primer, 5’-ATTA ATGAGCTCGTGAAAGGGCTGGA-3’ and reverse primer, 5’-ATTAATGAGCTCGG CGTATTATTCAGC-3’ was used. The sequence from all PCR derived constructs was confirmed by automatic sequencing.

### *PIK3IP1* promoter luciferase assay

293T were seeded into 24-well plates one day before transfection. Cells were transfected with either K-Ras expression or K-Ras empty plasmids and pGL4.10-PIK3IP1promWT, series deletion constructs or pGL4.10-PIK3IP1promWT with enhancer and control Renilla luciferase expression vector using Lipofectamine 2000 (Invitrogen) according to manufacturer’s instructions. Luciferase activity was assessed 24 h after transfection with the Dual-Luciferase Reporter Assay System (Promega). Transfections were performed at least in triplicate on three separate experiments. Luciferase signals were first normalized to Renilla. The relative amount of luciferase activity in the empty vector (pGL4.10) transfected cells was set to 1 and the values in other expression vectors were normalized to the empty vector.

### Western blot analyses

Total cell lysates were prepared with RIPA buffer (Thermo Scientific, Waltham, MA, USA), protease inhibitor mix (Complete Mini, Roche, Basel, Switzerland) and phosphatase inhibitor (PhosphoStop, Roche), followed by the addition of 40 ul of 1X sample buffer and boiled. The samples were subjected to SDS-PAGE and assayed by western blot using the corresponding antibodies. The following primary antibodies were used for immunoblot: anti-β-tubulin (T8328, Sigma), anti-p-ERK (#4370, CST, Danvers, MA, USA), anti-LSD1 (ab17721, Abcam), and anti-p-LSD1 (S131) (#37177, CST). The secondary antibodies used were anti-mouse IgG HRP and anti-rabbit IgG HRP (1:2000 dilution, Amersham, Little Chalfont, UK). Immunoreactive proteins were visualized using ECL reagent (Amersham).

### λ-phosphatase assay

After BJ-H-RasV12-ER cells were treated with 4-HT for 24 hr, they were then lysed with RIPA buffer with protease inhibitor mix and phosphatase inhibitor. After separating the lysates into two tubes, 400 units of λ-phosphatase (NEB, Ipswich, MA, USA) were added in either lysate and incubated for 10 min at 30 °C. The reactions were stopped with 50 mM EDTA and 5X sample buffer, and boiled for 10 min. The reaction mixture was resolved by SDS-PAGE and analysed by immunoblot with anti-LSD1 and anti-p-LSD1 (S131) antibodies.

### Colony formation assay

Two milliliter of 1% agar in DMEM containing 10% FBS was added to 6-well plates for the bottom agar. Five thousand A549/Tet-On/PIK3IP1 cells in DMEM containing 10% FBS and 0.8% agar were added to the bottom agar. The agar was topped up with 10% FBS/DMEM with or without Dox (1.25 µg/ml). The top media was replaced every other day. After 2–3 weeks, colonies were stained with 500 μl of 5 mg/ml MTT (#5655, Sigma) at 37 °C for 1 h and analysed using GelCount^TM^ (OXFORD OPTRONIX, Abingdon, UK).

### Statistical analysis

Each experiment was performed at least three times. The data are plotted as means ± SD. Statistical analysis was performed using two-sided Student’s *t*-test (*p < 0.05, **p < 0.01, ***p < 0.001 and ns not significant). A *P*-value of <0.05 was considered to indicate statistical significance.

## Supplementary information

Supplementary Information
