# Supplementary material for: A Ras-LSD1 axis activates PI3K signaling through PIK3IP1 suppression
Source: Oncogenesis. 2020 Jan 2;9(1):2. doi: 10.1038/s41389-019-0185-4 (PMC6949251; doi:10.1038/s41389-019-0185-4)
Supplement: Supplementary file 1 — Supplementary Information [file 41389_2019_185_MOESM1_ESM.docx]

**Supplementary Information**

**Supplementary Figures**





**Figure S1. 1 Identification of a 5′ minimal regulatory region of *PIK3IP1***

Analysis of 5’-deletion constructs revealed that the 5’ regulatory region is essential to drive the maximal promoter activity, with clear impact of the −250 bp to −200 bp region upstream of the transcription start site (TSS). This was further confirmed by additional deletions that resulted in a marked loss of promoter activity. To identify the minimal core *PIK3IP1* promoter region, we created a series of truncated promoter constructs contained -250/ -200, -250/-150 and -250/-100. The 150 bp sequence located at -250/-100 bp of the TSS had maximum promoter activity and was required for the basal promoter activity of the *PIK3IP1* gene**.** For preparation of pGL4.10 (-250/-200), (-250/-150) and (-250/-100), reverse primers sequences used were:

5’-CCCCTGGGTCCTAAGCCTCCTAGGTCATGCAA-3’ (-250/-200),

5’-CTTCCCAGCTAGCTTGCTGCAGCGCGAGCTGT-3’ (-250/-150),

5’-TGCCCTTGTTATGCTGTTCTGGTAAACAGCCT-3’ (-250/-100).

One forward primer, 5’-TCTTGGCTCAGTTAGATTTTGCATGACCTAGG-3’ was used. All PCR products were digested with SacI and XhoI and cloned into pGL4.10 [luc2] (Promega).


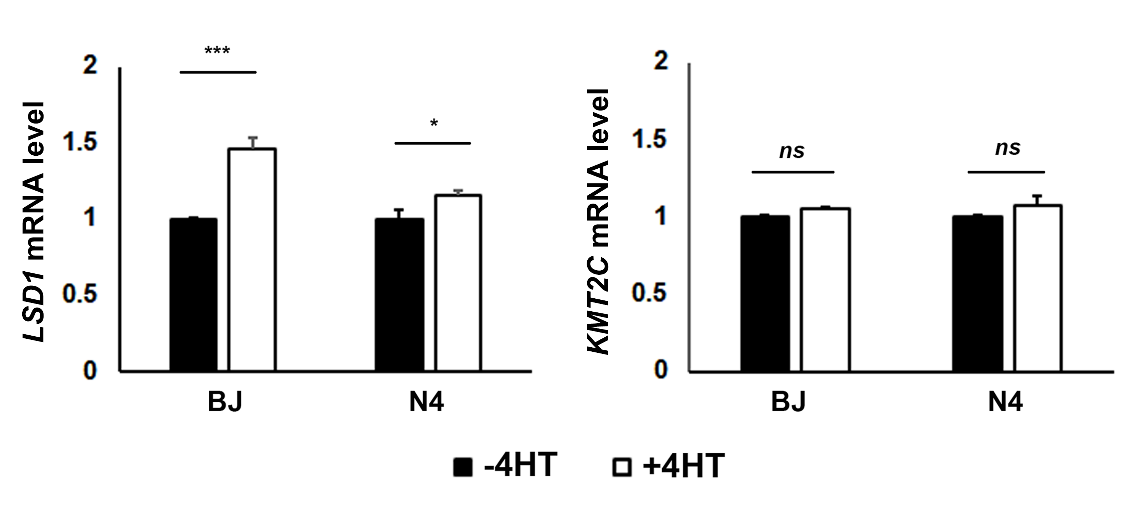


**Figure S2.**  ***LSD1* (Lysine demethylase) not *KMT2C* (Lysine methyl transferase 2C) mRNA expression increases upon Ras activation**

Relative *LSD1* (Right) and *KMT2C* (Left) mRNA abundance in BJ-H-RasV12-ER and N4-H-RasV12-ER cells was assessed by qPCR 24 hr after treatment with 4-HT.

qPCR analysis was performed using the iQ SYBR Green Super mix (BIO-RAD) with the following gene-specific primers: human LSD1 (FW 5’-CAGGCTTGGCAGCAGCTCGA-3’, REV 5’-TCCACCCACACGATCCCTGGC-3’) and human KMT2C (FW 5’-CCACGAA AACAAAGAGGACAG-3’, REV 5’-TGGGTGCTTACACTTACACAAGAT-3’) and The TATA-binding protein (TBP) gene was used for normalization. All PCR reactions were performed in triplicate.


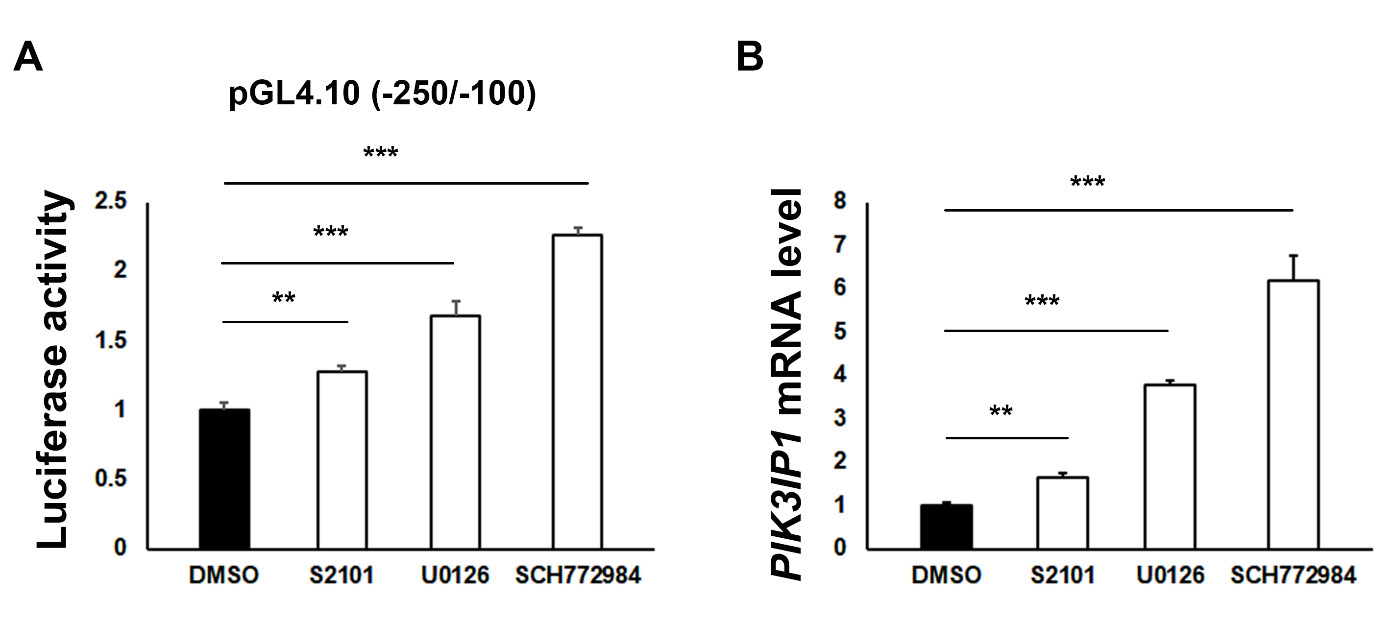


**Figure S3. LSD1 or MEK/ERK inhibition significantly increased *PIK3IP1* promoter activity and mRNA expression level in the absence of RAS overexpression.**

**A** *PI3KIP1* promoter activity regulated by inhibitors of MEK, ERK and LSD1. 293T cells were co-transfected with pGL4.10-Luc (-250/-100), minimal regulatory region of promoter, and K-Ras empty plasmids. 24 hr after transfection, co-transfected cells were treated with the indicated compounds. After 8 hr, the cells were collected and assayed for luciferase activity. Values were normalized against luciferase activity of DMSO treated cells. The experiments were performed in triplicate.

**B** Relative *PIK3IP1* mRNA expression by qPCR analysis in BJ-H-RasV12-ER cells were treated the indicated compounds without 4-HT for 24 hr. PIK3IP1 expression with DMSO was set to 1.

Inhibitors and their concentrations used: LSD1 inhibitor (S2101, 20 μM), MEK inhibitor (U0126, 10 μM) and ERK inhibitor (SCH772984, 1 μM). Data were presented as the means ± SD of three independent experiments. **p < 0.01 and ***p < 0.001 by Student’s t-test as compared to control cells


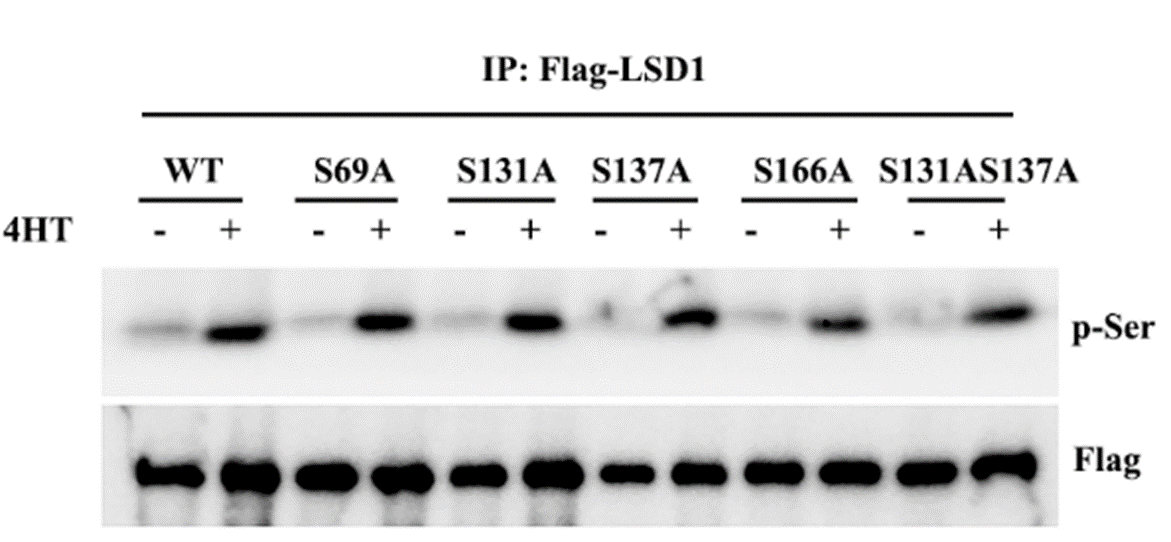


**Figure S4. LSD1 activity is not abrogated by the mutation of multiple putative phosphorylation sites of LSD1.**

IP assay of wild-type and at S69A, S131A, S137A, S166A and S131AS137A mutant FLAG-tagged LSD1-transfected N4-H-RasV12-ER cells treated with (+) or without (-) 4HT.

Total cell lysates were prepared with RIPA buffer (Thermo Scientific, Waltham, MA, USA), protease inhibitor mix (Complete Mini, Roche, Basel, Switzerland) and phosphatase inhibitor (PhosphoStop, Roche). The Co-IP analyses were performed using ~500 µg protein samples that were incubated with either 1 ug of IgG or anti-FLAG (PM020, MBL, Japan) for one hr at 4°C. In total, 20 µl Protein A/G PLUS-Agarose (sc-2003, Santa Cruz**,** Santa Cruz, CA, USA) were added, and the samples were incubated at 4°C overnight. The pellets were then washed three times with 1.0 ml PBS, followed by the addition of 40 ul of 1X sample buffer and boiled. The samples were subjected to SDS-PAGE and assayed by western blot using the anti-phosphorylated serine (p-Ser) (AB1603, Merck, Darmstadt, Germany).





**Figure S5.** **LSD1 inhibition in combination with Ras pathway increases apoptosis in Ras/Raf mutated cancer cells**

**A** A549 cells were treated either S2101 (LSD1 inhibitor), SCH772984 (ERK inhibitor) or S2101 together with SCH772984 for 48 hr. The morphological feature of apoptosis such as shrinkage of the cells have been increased in SCH772984 together with S2101 treated A549 cells compared to only SCH772984 treated cells.

**B** The western blotting with anti-capase-3 antibody also showed that LSD1 inhibition in combination with ERK inhibition strongly increased cellular apoptosis in Raf-mutated HT29 cells as wells as Ras-mutated A549 cells. β-tubulin was used as a loading control
